# Lung adenocarcinomas with isolated *TP53* mutation: A comprehensive clinical, cytopathologic and molecular characterization

**DOI:** 10.1002/cam4.6873

**Published:** 2024-01-02

**Authors:** Rachelle P. Mendoza, Heather I‐Hsuan Chen‐Yost, Pankhuri Wanjari, Peng Wang, Emily Symes, Daniel N. Johnson, Ward Reeves, Jeffrey Mueller, Tatjana Antic, Anna Biernacka

**Affiliations:** ^1^ Department of Pathology University of Rochester Medical Center Rochester New York USA; ^2^ Department of Pathology University of Michigan Ann Arbor Michigan USA; ^3^ Department of Pathology The University of Chicago Hospitals Chicago Illinois USA; ^4^ Department of Pathology OSF Little Company of Mary Medical Center Evergreen Park Illinois USA

**Keywords:** isolated *TP53* mutation, lung adenocarcinoma, lung cancer

## Abstract

**Background:**

*TP53* mutation is present in about 50.8% of lung adenocarcinomas, frequently in combination with other genetic alterations. However, a rare subset harbors the *TP53* mutation alone.

**Methods:**

Next‐generation sequencing was performed in 844 lung adenocarcinomas diagnosed by fine needle aspiration. Fourteen cases (1.7%) showed isolated *TP53* alteration and were subjected to a comprehensive analysis.

**Results:**

The average age at diagnosis was 65.7 years (range 48–79); 9 males and 5 females. All were smokers with an average pack‐year of 40.7 (range 10–70). Ten had metastases, mostly in the brain (*n* = 4) and pleura (*n* = 4). After a follow‐up period of up to 102 months, 9 died, 3 were alive free of disease, 1 was alive with disease, and 1 was lost to follow‐up. The median survival was 12.2 months. Most tumors exhibited poor differentiation, composed of solid sheets with moderate to severe atypia, increased mitotic activity, and necrotic background. Half were positive for TTF‐1 and showed p53 overexpression. PD‐L1 was positive in 5 cases. Most alterations were missense mutations in exons 5–8, and this mutation type was associated with p53 overexpression. Tumors with combined missense mutation and truncated protein had higher PD‐L1 expression along with a trend towards an increase in tumor mutational burden (TMB). *CEBPA* deletion of undetermined significance was the most common copy number alteration.

**Conclusion:**

Isolated *TP53* mutation was seen in association with smoking, high‐grade cytomorphologic features, adverse prognosis, and recurrent *CEBPA* deletions. These tumors tend to have strong PD‐L1 expression and high TMB, suggesting potential benefit from immune checkpoint inhibitors. Hence, the recognition of this molecular group has prognostic and therapeutic implications.

## 1 INTRODUCTION

Non‐small cell lung carcinomas (NSCLC) have a heterogeneous histologic and molecular profile. Molecular analysis has become an essential component in lung adenocarcinoma workup due to the advent of targeted small molecule monoclonal antibody treatment. Common driver mutations that have targeted therapies are *EGFR*, *KRAS*, and *ALK*.^1^ Some NSCLCs have *TP53* mutations, either in combination with other driver mutations or in isolation. *TP53* mutation is present in about 23% to 65% of NSCLC and can be seen in up to 50.8% of lung adenocarcinomas.^2^, ^3^, ^4^ The majority of *TP53* mutations occur in a hotspot, which is the DNA‐binding region in exons 5–8.^5^ The mutations are typically missense or nonsense mutations, leading to loss of activity.^5^ NSCLCs with concurrent *TP53* mutation have worsened survival and poorer response to chemotherapy and radiation.^6^ While a subset of lung adenocarcinomas with sole *TP53* mutation exhibits high‐grade fetal lung‐like morphology,^7^ there were rare cases of NSCLC with usual adenocarcinoma morphology that harbor *TP53* mutation alone as a molecular alteration.

*TP53* alterations have been described in small cell lung carcinoma (>90%),^6^ squamous cell carcinoma (81%),^8^, ^9^ and lung adenocarcinomas (40%–46%), chiefly in association with other driver mutations and in current or former smokers.^8^, ^9^ In literature, all investigations on *TP53*‐mutated lung adenocarcinoma have been in the setting of other co‐mutations.^3^, ^6^, ^10^, ^11^ The clinical and diagnostic significance of an isolated *TP53* mutation in the absence of any other driver mutation has never been investigated. In this study, we aim to characterize lung adenocarcinomas with isolated *TP53* mutation with emphasis on clinical, cytomorphologic, and molecular features, providing the largest series to date on lung adenocarcinomas with isolated *TP53* mutations. We also aim to explore the clinical and molecular profile of each *TP53* mutation type and its subsequent effect on the function of the p53 protein.

## 2 METHODS

### 2.1 Study population

Lung adenocarcinoma cases consecutively diagnosed between January 2016 and January 2021 by endobronchial ultrasound‐guided transbronchial needle aspiration biopsies were retrospectively reviewed. Molecular sequencing performed as part of the diagnostic workup was evaluated, and tumors showing pathogenic *TP53* alterations alone were included in the study. Tumors harboring additional pathogenic genomic alterations involving other genes were excluded. This study was approved by the University of Chicago Institutional Review Board (IRB18‐1438). A waiver for obtaining written informed consent was granted by the Institutional Review Board on the basis of including only previously collected material in this study.

### 2.2 Pathologic analysis

The following cytomorphologic features were analyzed for each case: background, architecture, nuclear size, nuclear‐to‐cytoplasmic ratio, pleomorphism, chromatin quality, nuclear membrane contour, and intranuclear inclusions. Each cytologic specimen was reviewed by three authors (AB, TA, RM). Concurrent histopathologic specimens, if available, were recorded as originally diagnosed and characterized by experienced thoracic pathologists. Cytologic grading was performed using the parameters discussed by Sigel et al., including smear background, cellular arrangements, presence of giant tumor cells, nuclear size, and nuclear contour.^12^

### 2.3 Immunohistochemistry

Immunohistochemistry (IHC) was performed on the cell block samples and/or concurrent core biopsy/surgical resection specimen. The IHC studies performed included: TTF‐1 (clone 867G311, Dako, Carpinteria, CA), SALL4 (clone EE‐30, Santa Cruz, Dallas, Texas), CDX2 (clone AMT28, Novocastra, Leica Biosystems, Buffalo Grove, IL), CK7 (clone OV‐TL, Dako, Carpinteria, California), CK20 (clone K_s_20.8, Dako, Carpinteria, CA), synaptophysin (clone 27G12, Leica, Buffalo Grove, IL), chromogranin (clone LK2H10, ThermoFisher Scientific, Pittsburgh, PA), p53 (clone DO‐1, Calbiochem, Temecula, CA), and PD‐L1 (clone 22C3 pharmDx, Dako, Carpinteria, CA). PD‐L1 expression was determined using tumor proportion score as described in the manufacturer's interpretation manual. All IHCs were clinically validated for use in alcohol‐fixed and formalin‐fixed specimens and performed at a dilution of 1:50 using validated protocols on either Leica BOND‐III (Leica Biosystems, Buffalo Grove, IL) or BenchMark X.T. Ventana platforms (Roche, Tucson, AZ).

### 2.4 Next‐generation sequencing

DNA Next‐generation sequencing (NGS) was performed using the University of Chicago Medicine OncoPlus (UCM‐OncoPlus) panel with a representative Diff‐Quik stained cytology smear or formalin‐fixed paraffin‐embedded tumor block. UCM‐OncoPlus is a hybrid‐capture panel targeting 1005 cancer‐associated genes with 168 clinically reported genes, as previously described.^13^ Somatic mutation calling was performed across all genes using a custom in‐house bioinformatics pipeline as previously described.^13^ The variant review was performed by two of the authors (PW, PW) and included filters based on population variant frequencies (The 1000 Genomes Project, https://www.internationalgenome.org/), variant frequencies in cancer databases (COSMIC: catalogue of somatic mutations in cancer, https://cancer.sanger.ac.uk/cosmic and cBioPortal, https://www.cbioportal.org/), and coding effects. Somatic variant calls were inspected using Integrated Genomics Viewer (IGV; Broad Institute, MIT Harvard, Cambridge, MA). Copy number results were calculated using a combination of CNVkit^14^ software and additional in‐house intrarun normalization to eliminate run‐specific artifacts by comparison with a pooled cohort of clinical controls.^15^ Gene‐level changes were called using the UCM‐OncoPlus clinical interpretation criteria as previously described.^13^ The International Agency for Research on Cancer (IARC) *TP53* Database (World Health Organization, Lyon, France) was used to determine the characterization of the activity of the *TP53* mutation in each tumor.

RNA sequencing was performed with the University of Chicago's RNA Oncoplus Assay for gene fusion analysis, which is a hybrid capture‐based RNA sequencing assay for detecting known and novel fusions involving any of the 1005 targeted cancer‐associated genes. RNA was extracted using the simplyRNA Tissue Kit on Maxwell RSC® instrument (Promega, Madison, WI) and quantified using a Qubit fluorometric assay (Thermo Fisher Scientific, Foster City, CA), adjusted for the percentage of fragments greater than 100 bp using a TapeStation system (Agilent, Santa Clara, CA). In total, 300 ng of RNA was subjected to library prep using the KAPA Stranded RNA‐Seq Kit with RiboErase (Kapa Biosystems, Wilmington, MA), followed by quantitation using the KAPA library quantification kit (Kapa Biosystems, Wilmington, MA). Pooled libraries were captured using a panel of biotinylated oligonucleotides (xGen Lockdown probes, Integrated DNA Technologies, San Diego, CA). Amplified pooled captured libraries were sequenced in rapid run mode on a NovaSeq 6000 system (Illumina, San Diego, CA) to produce 2 × 101 bp paired‐end sequencing reads. Sequence data were aligned to the hg19 human reference transcriptome using STAR aligner (*Bioinformatics 2013; 29(1): 15–21*), and fusions were detected using a combination of in‐house developed Python software and STAR fusion software.

### 2.5 Statistical analysis

The demographics, cytomorphology, immunohistochemistry, and molecular information were analyzed descriptively. The associations between molecular profiles, clinical, cytomorphologic, and immunohistochemical features were performed using Chi‐square or Fisher's exact test, whichever was appropriate, for categorical variables. Mann–Whitney *U* or Kruskal–Wallis *H* test was utilized for continuous variables. All hypothesis tests were two‐sided, and statistical significance was set at *p* < 0.05. All statistical analysis was performed using IBM SPSS version 29 (Chicago, IL).

## 3 RESULTS

### 3.1 Demographics

Out of 844 lung adenocarcinomas diagnosed by cytology within a 5‐year period, 14 cases (1.7%) with sole *TP53* mutation were identified. Table 1 summarizes the clinical features of these patients. The average age of the patients at diagnosis was 65.7 years (range 48 to 79), consisting of nine males (64.3%) and five females (35.7%). All the patients were tobacco smokers with an average pack‐year of 40.7 (range 10 to 70 pack‐years). Most patients presented with respiratory symptoms such as cough (*n* = 6), worsening dyspnea/shortness of breath (*n* = 6), and hemoptysis (*n* = 3). Two patients had unrelated symptoms, and the lung masses were incidentally discovered during imaging for extremity swelling (*n* = 1) and hematuria with right flank pain (*n* = 1). Most patients were diagnosed at clinical stage IV (*n* = 5), followed by IIIA (*n* = 3) and IIIB (*n* = 2). Those with stage IV disease had metastatic lesions in the brain (*n* = 2), pleura/pleural effusion (*n* = 2), contralateral lung (*n* = 1), distant organs (*n* = 2), and distant lymph nodes (*n* = 2). The average primary tumor size was 3.7 cm (range 1.3 to 8.1 cm), eight (57.1%) of which were found in the right lung, and six (42.9%) were left‐sided.

**TABLE 1:** Clinical characteristics of patients diagnosed with lung adenocarcinoma with isolated *TP53* mutation.

Case	Sex	Age	Pack years	Location	Laterality	Tumor size (cm)	Clinical stage at diagnosis	Treatment	Recurrence	Metastasis	Status at follow‐up	RFS (months)	OS (months)
1	M	70	50	Hilum	R	6.4	IV	Refused	No	Brain, adrenal, distal nodes	LTFU	Unknown	Unknown
2	M	72	30	Hilum	R	2.7	IV	C	No	Supraclavicular node	DOD	6.0	6.0
3	M	78	30	Lower lobe	L	3.6	IIIA	S + C + Ra	Yes	Brain, pleura	DOD	6.2	9.8
4	F	70	50	Lower lobe	L	4.9	IV	C + Ra	Yes	Pelvis, paracolic gutter, omentum, clavicle	DOD	29.4	34.1
5	M	62	45	Upper lobe	L	8.1	IIIA	S + C + Ra (brain only)	Yes	Brain	Alive, NED	12.5	52.2
6	M	69	50	Lower lobe	L	2.2	IIIB	C + Ra	Yes	Rectal area	DOD	11.8	12.2
7	F	72	20	Hilum	L	4	IV	C	Yes	Chest wall, diaphragm, pleura, ribs	DOD	2.6	6.3
8	M	53	35	Upper lobe	R	1.4	IIIB	Refused	Yes	Omentum	DOD	8.5	8.5
9	M	79	50	Upper and lower lobes	R	3	IIA	Refused	Unknown	Unknown	DOD	8.3	8.3
10	M	48	30	Lower lobe	R	5.3	IIB	S + C	No	None	Alive, NED	64.3	64.3
11	F	65	10	Perihilar	L	1.5	IV	S + C + Ra	Yes	Brain, pleura, nodes	AWD	0.0	68.8
12	F	67	40	Upper lobe	R	1.3	Unknown	Refused	No	Unknown	DOD	3.3	3.3
13	F	60	60	Lower lobe	R	6.4	IIIA	C + Ra	Yes	Pleura, paramediastinal	DOD	84.8	101.4
14	M	55	70	Hilum	R	1.5	IA1	S + C	No	None	Alive, NED	36.6	36.6

Abbreviations: AWD, alive with disease; C, chemotherapy; DOD, died of disease; L, left; LTFU, lost to follow‐up; NED, no evidence of disease; OS, overall survival; Ra, radiotherapy; R, right; RFS, recurrence‐free survival; S, surgery.

After diagnosis, 10 patients received treatment, while four declined any form of treatment. All ten patients who received treatment had chemotherapy (71%); the most common regimen was a combination of platinum‐based therapy (cisplatin, carboplatin) and pemetrexed (*n* = 7). Four individuals received immune checkpoint inhibitors, including pembrolizumab (*n* = 3) and ipilimumab/nivolumab (*n* = 1) in combination with platinum‐based chemotherapy. Six received radiotherapy, one of which was brain only. Five had surgical resection of the lung tumor, two of whom developed local recurrence 6.2 and 84.8 months post‐surgery. Four other patients had recurrences: two in the pleural fluid, one in the brain, and one in the peritoneum/omentum. The median progression‐free survival was 8.4 months (range 0 to 84.8 months). After a variable follow‐up period ranging from 3 months to 8 years, 9 patients died of disease, 3 were alive without disease, 1 was alive with stable disease, and 1 was lost to follow‐up. The median overall survival was 12.2 months (range 3.3 to 101.4 months).

### 3.2 Cytomorphology

**FIGURE 1:**
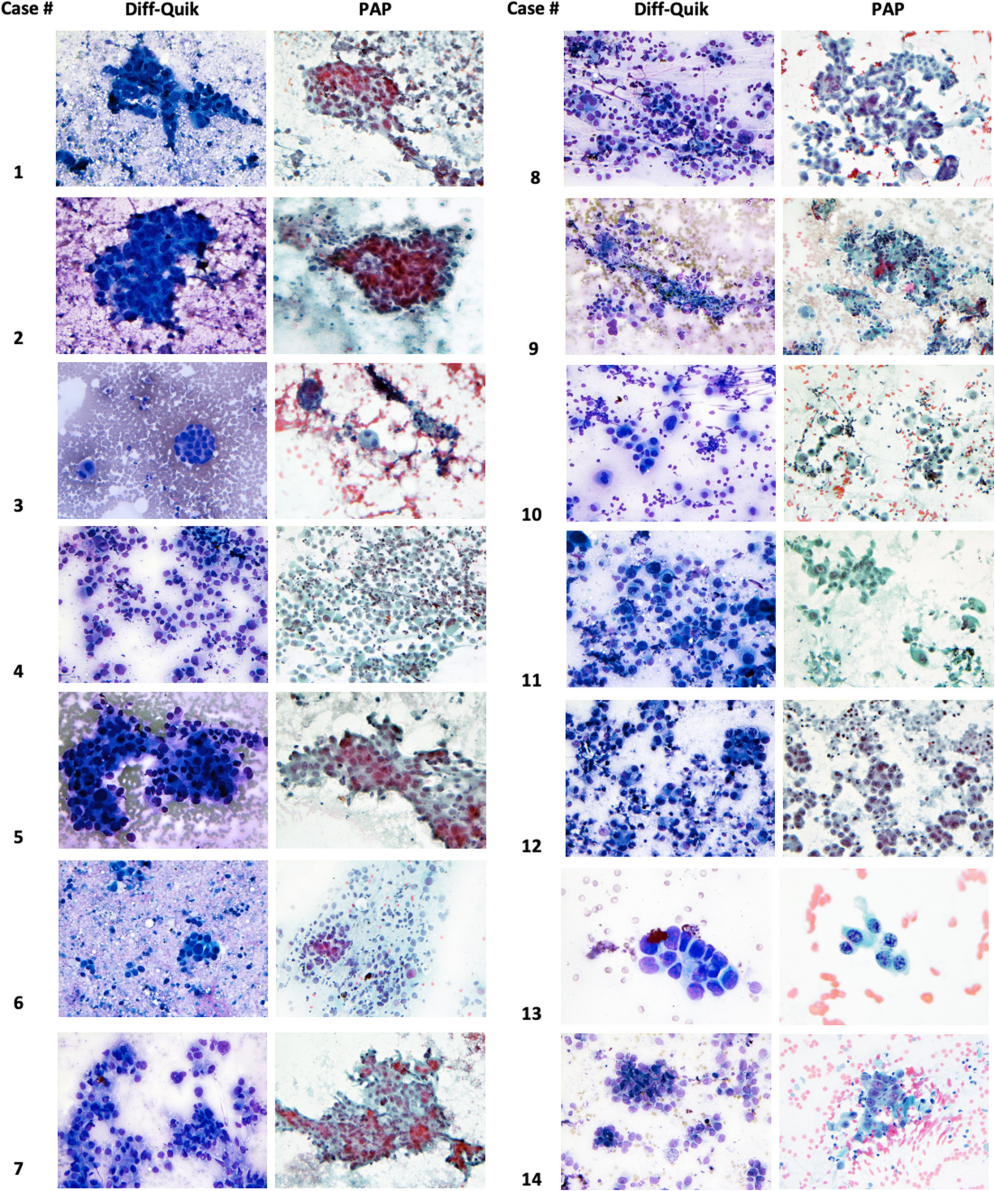
Cytomorphology (Diff‐Quik and Papanicolaou stains) of lung adenocarcinomas with isolated *TP53* mutation. Ten tumors (cases 1, 2, 4, 5, 8, 9 10, 11, 12, 14) were poorly differentiated, demonstrating sheets and clusters of tumor cells, while four (cases 3, 6, 7, 13) showed moderate differentiation with foci of acinar and glandular formation. Most lesions (cases 1, 2, 4, 6, 8, 9, 10, 11, 12, 13, 14) showed mild to moderate chronic inflammatory background, while a subset (cases 1, 6, 11) demonstrated necrotic backgrounds. None of the cases displayed fetal lung‐like morphology or architecture. All tumors demonstrated a high nuclear‐to‐cytoplasmic (N:C) ratio, hyperchromatic enlarged nuclei, and moderate to marked nuclear pleomorphism. The tumor cells displayed uneven, coarsely granular chromatin, irregular nuclear membrane, and rare intranuclear inclusions. Mitotic figures were frequently identified.

The cytomorphologic features of the cases are shown in Figure 1. Ten tumors were poorly differentiated, while four showed moderate differentiation. Most cases (*n* = 7) displayed sheets and clusters of atypical cells. Four tumors had foci of acinar and glandular formation, with rare cells showing intracytoplasmic mucin. Single, markedly atypical tumor cells were identified in all cases. Most lesions (*n* = 11) showed mild to moderate chronic inflammatory background, while a subset (*n* = 3) demonstrated necrotic backgrounds. None of the cases displayed fetal lung‐like cytomorphology or architecture. All tumors demonstrated a high nuclear‐to‐cytoplasmic (N:C) ratio, hyperchromatic enlarged nuclei, and moderate to marked nuclear pleomorphism. The tumor cells displayed uneven, coarsely granular chromatin, irregular nuclear membrane, and rare intranuclear inclusions. Mitotic figures were frequently identified. The cytomorphologic characteristics correlated with the predominant histologic features in the biopsy and/or resection specimen.

### 3.3 Immunohistochemical studies

As expected, all cases were positive for CK7 (*n* = 14), and most were negative for CK20 (*n* = 11). One case showed strong CK20 expression, while two cases had patchy staining. TTF‐1 was positive in only 50% of the cases, while two tumors showed CDX2 expression. One case displayed focal expression of synaptophysin and chromogranin. All cases were negative for SALL4. Increased nuclear p53 expression (>70% of tumor nuclei) was observed in 7 tumors (50%), while the rest showed weak, patchy nuclear staining (wild‐type). PD‐L1 was positive in 5 cases (35.7%), showing moderate to strong expression in 50%–100% of tumor cells.

### 3.4 Molecular analysis

NGS was performed on either lymph node metastasis (*n* = 8) or the primary lung tumor (*n* = 6). The tumors displayed varying types and locations of *TP53* mutation, most of them located in exons 5–8 (*n* = 8). While most were characterized by the IARC *TP53* Database as missense mutations (*n* = 8), some were nonsense (*n* = 2), frameshift (*n* = 2), and deletion or insertion–deletion mutations (*n* = 2). Most mutations (*n* = 7) were transversions (a purine is changed to a pyrimidine, or vice versa). These mutations commonly led to negative effects on p53 function, including loss of function or reduced activity (*n* = 11), and dominant negative (*n* = 1). Interestingly, one tumor mutation had a gain of function effect, and one deletion in exon 6 had unknown activity (*n* = 1). Most mutations were located in exon 5 of the *TP53* gene (*n* = 5), followed by exon 4 (*n* = 3) and exon 8 (*n* = 2). One tumor showed mutations in exons 2, 3, and half of 4.

The mean tumor mutational burden (TMB) was 11.7 mut/Mb (range 2.1 to 38). Using the institutional cut‐off of 10 mut/Mb for high TMB, eight tumors (57.1%) had low TMB, and six (42.9%) had high TMB. All tumors were microsatellite stable, with the mean microsatellite instability (MSI) score at 3.7 (range of 1.8 to 8.0).

Copy number variations of undetermined significance (VUS) were observed in nine tumors, six of which had both amplifications and losses (Table 2). Eight tumors had amplifications in 1 to 11 genes (average 4.1). While there is no recurrent gene amplification that was observed, the following genes were amplified in two tumor samples: *MET, SMO, ERBB2, BRAF, EZH2, SRSF2, CUX1*, and *CEBPA*. Seven tumors had gene loss, and the number of genes with losses ranged from 1 to 4 (average 2). Notably, five tumors showed loss of *CEBPA* (chromosome 19). Other frequent genes with copy number loss were *RAD51* (*n* = 2) and *DDX41* (*n* = 2).

**TABLE 2:** Molecular profile of lung adenocarcinoma with isolated *TP53* mutation.

Case	Specimen site	Allele frequency	*TP53* mutation					Tumor mutational burden		Microsatellite status		Copy number variation	
			*TP53* pathogenic variants	Location	Type of mutation	Effect	Amino acid change	TMB	TMB Low/High	MSI Score	MSI status	Gain	Loss
1	Lymph node	76%	c.464C > A, p.T155N	Exon 5	Missense	Dominant negative	Threonine to asparagine	21.5	High	7.5	MSS	*EGFR, GRIN2A, ERBB2*	*DDX41, CDKN2A, SMAD4, CEBPA*
2	Lymph node	74%	c.884del, p.P295Lfs*50	Exon 8	Frameshift	Reduced activity	‐	7.4	Low	2.4	MSS	*GATA2, FOXL2, CUX1, MET, SMO, BRAF, EZH2, MYC, SRSF2*	*RAD51, CEBPA*
3	Lung	25%	c.298C > T, p.Q100*	Exon 4	Nonsense	Reduced activity	‐	4.5	Low	1.8	MSS	*ERBB2, SRSF2*	*CEBPA*
4	Lung	16%	c.716A > C, p.N239T	Exon 7	Missense	Loss of function	Asparagine to threonine	10.5	High	1.8	MSS	*MET7*	*CEBPA*
5	Lymph node	70%	c.193A > T, p.R65*	Exon 4	Nonsense	Reduced activity	Arginine to stop	38.4	High	3.3	MSS	*RAD21*	*PIK3R1, DDX41, FGFR1, HRAS*
6	Lymph node	65%	c.473G > T, p.R158L	Exon 5	Missense	Loss of function	Arginine to leucine	10.5	High	2.4	MSS	*PIK3CA, TERT, BIRC3, ATM, CEBPA*	*RAD51*
7	Lymph node	72%	c.313G > T, p.G105C	Exon 4	Missense	Partial loss of function	Glycine to cysteine	4.2	Low	8.0	MSS	*FBXW7, CDK6, CUX1, MET, SMO, BRAF, EZH2, CEBPA, AXL, MAPK1, SMARCB1*	None
8	Lung	6%	c.839G > C, p.R280T	Exon 8	Missense	Gain of function	Arginine to threonine	2.1	Low	3.0	MSS	None	*CEBPA*
9	Lung	25%	c.‐28‐597_233del, p.0?	Exon 2, 3, half of 4	Deletion	Reduced activity	‐	3.9	Low	5.2	MSS	None	None
10	Lung	15%	c.623_634del, p.D208_F212delinsV	Exon 6	Deletion	Unknown	In‐frame deletion with valine substitution	7.4	Low	1.8	MSS	None	None
11	Lymph node	24%	c.461G > T, p.G154V	Exon 5	Missense	Loss of function	Glycine to valine	3.2	Low	3.0	MSS	None	None
12	Lymph node	71%	c.956del, p.K319Rfs*26	Exon 10	Frameshift	Reduced activity	‐	17.1	High	5.4	MSS	*HNF1A*	None
13	Lymph node	39%	c.380C > T, p.S127F	Exon 5	Missense	Loss of function	Serine to phenylalanine	31.7	High	3.9	MSS	None	None
14	Lung	4%	c.476C > T, p.A159V	Exon 5	Missense	Loss of function	Alanine to valine	2.1	Low	3.0	MSS	None	None

Abbreviations: MSI, microsatellite instability; MSS, microsatellite stable; TMB, tumor mutational burden.

### 3.5 Correlations

Overexpression of p53 was associated with a missense type of mutation (*p* = 0.003), as 6 of 7 tumors with p53 overexpression by IHC had *TP53* missense mutation (Table S1). In addition, tumors with missense mutation and truncated protein were associated with a higher proportion of PD‐L1 expression (*p* = 0.035) (Figure S1A).

There was no significant correlation between the type of *TP53* alteration and TMB (*p* = 0.533 and 0.573 for categorical and continuous data, respectively). The tumors with missense mutations had an average TMB of 7.3 mut/Mb (range 2.1 to 31.7), while those with non‐missense had an average TMB of 13.1 mut/Mb (range 3.9 to 38.4). However, when categorized according to the subsequent effect of the mutation, the tumors with truncated p53 protein due to missense mutation had a higher average TMB (17.4 mut/Mb) than those with non‐missense mutation (12.2 mut/Mb) (Figure S1B). The same pattern was seen in tumors with altered DNA‐binding domain—those with missense mutation had higher mean TMB (8.5 mut/Mb) than those with non‐missense alterations (5.9 mut/Mb).

## 4 DISCUSSION

This study characterized the clinical, cytomorphologic, immunohistochemical, and molecular profile of lung adenocarcinomas with isolated *TP53* mutations. All patients were smokers, and most had metastatic disease at presentation and later died of the disease. The cytomorphology was poorly differentiated in most of the tumors, while a subset showed foci of obvious glandular differentiation. All cases showed high‐grade tumor morphology with marked nuclear enlargement and pleomorphism, high N:C ratio, and increased mitotic activity. Overexpression of p53 and TTF‐1 positivity were observed in only half of the cases, while PD‐L1 was strongly positive in over a third of these tumors. Most tumors had missense *TP53* mutations, and the missense mutation type was correlated with p53 overexpression. Missense mutations leading to a truncated p53 protein effect were associated with PD‐L1 positivity and higher TMB. Recurrent loss of *CEBPA* was observed in the tumor cohort.

*TP53* alterations have been described in lung cancers, with the highest prevalence in small cell (>90%)^6^ and squamous cell carcinoma (81%)^8^, ^9^; both subtypes are most consistently associated with long‐term smoking. Mutations in *TP53* have been identified in approximately 40%–46% of lung adenocarcinomas, chiefly in association with other driver mutations and in current or former smokers.^8^, ^9^ The frequency and types of *TP53* alterations in lung adenocarcinomas differed between smokers and never smokers.^3^, ^16^ In fact, smokers had a 3–4 times increased risk of acquiring a *TP53* mutation in comparison to non‐smokers.^17^, ^18^ The association between smoking and *TP53* mutation is further supported by the clinical profile of the present study cohort, all of whom were former or current smokers. In addition, the most frequent alteration in these tumors involves transversion mutations, a known base change in smokers and strongly correlated to exposure to the carcinogens found in tobacco.^3^, ^9^, ^19^

While most studies suggest that lung adenocarcinomas with *TP53* mutations carry a worse prognosis and are more resistant to chemotherapy and radiation,^3^, ^10^, ^20^, ^21^, ^22^, ^23^ others have reported equivocal prognostic value with respect to *TP53* mutation status.^11^, ^19^, ^24^ A study by Zhao et al. developed a p53‐deficiency score based on a transcriptomic profile and considered differential gene signatures found in p53‐deficient lung adenocarcinomas.^25^ Their results showed that the p53‐deficiency score was a predictor for recurrence‐free survival, and a high score was associated with poor survival. The association of p53 deficiency with transcriptomic alterations and subsequent poor clinical outcomes underscores the important role of p53 in the regulation of multiple genetic pathways. Recent investigations also reveal that p53 mutations can lead to abnormalities in microRNAs and epigenetic changes.^20^ The present cohort of lung adenocarcinomas further establishes evidence that carcinogenesis with poor clinical outcomes can occur secondary to *TP53* mutation alone. Most patients in this study were diagnosed at a late clinical stage and died of the disease, with shorter median recurrence‐free (8.5 months) and overall survival (12.2 months) compared to published data in the literature (12 months and 39 months, respectively).^26^

One recurrent molecular finding of undetermined significance in this cohort was the recurrent *CEBPA* losses in five tumors. CCAAT/enhancer binding protein alpha (*CEBPA)* alterations are more prevalent in hematologic malignancies,^27^ where *CEBPA* mutations have been associated with improved outcomes in pediatric and adult acute myeloid leukemia.^28^ In solid tumors, *CEBPA* alterations were most frequently observed in lung adenocarcinomas, consisting primarily of mutations followed by amplifications, missense, frameshift, insertion, and loss.^29^ When fully functional and overexpressed, the CEBPA transcription factor was shown in cell culture to inhibit migration and invasion of lung adenocarcinoma by suppressing epithelial‐to‐mesenchymal transition and the *Wnt*/beta‐catenin pathway.^30^ The frequent loss of *CEBPA* in *TP53*‐mutated lung adenocarcinoma may provide an important mechanism for the aggressive clinical behavior and associated poor prognosis of these tumors.

Most of the lung adenocarcinoma in the present study harbored a missense type of mutation in the *TP53* gene, and the dominant effect for the specimens was mostly loss of function or reduced activity. Loss of function of *TP53* has been well‐documented as an integral component of tumor progression in lung adenocarcinoma.^31^ The most common locations for the mutation were exon 5, followed by 4 and 8. Mutations in exon 5 have been shown to be associated with shorter survival, but mostly in lung squamous cell carcinomas.^32^ Exon 8 was also correlated with poor prognosis and nodal metastasis.^33^, ^34^ Interestingly, mutations in exon 4 of *TP53* have been shown to be a promising predictive and prognostic indicator but mostly for lung carcinomas with associated *EGFR* mutations.^35^ Of note, one case showed a mutation that acquires gain‐of‐function p53 activity. Such phenomenon has been reported in several cancer types, where a conformational change in p53 enables interaction with new transcription factors, such as p63, p73, NF‐Y, Sp1, NF‐κB, ATM, and SMADS, altering the transcription, cell cycle, apoptosis, and metabolism of cancer cells.^36^, ^37^ These mechanisms support the hypothesis that mutant p53 can promote tumorigenesis, cancer progression, metastasis, and even treatment resistance.^38^, ^39^

The cytomorphology of most of the tumors in the present study was poorly differentiated, and all cases showed high‐grade cytomorphologic features. A high prevalence of p53 mutations has been reported in poorly differentiated carcinomas^40^ and contributes to a stem cell‐like transcriptional signature.^41^ Wild‐type p53 has been reported to repress the expression of several cancer stem cell markers, including CD44, c‐Kit, NANOG, and OCT4.^42^ Loss of p53 function would lead to loss of this repression, resulting in a poorly differentiated morphology and enhanced chemoradiation resistance.^42^

Only half of the lung adenocarcinoma tumors in this study were positive for TTF‐1. While TTF‐1 expression is characteristic of lung adenocarcinoma, it should be noted that TTF‐1 is only positive in up to 80% of lung adenocarcinomas.^43^ Negative TTF‐1 expression in lung adenocarcinomas has been linked to worse prognosis.^44^, ^45^ The loss of TTF‐1 expression in the present study could be secondary to poor histologic differentiation due to underlying *TP53* mutations, as previously reported.^46^

Despite the detection of *TP53* alterations on NGS, only half of the tumors had aberrant nuclear p53 expression, and most of these had a missense type of mutation. Immunohistochemical stains for p53 have been deemed reliable in predicting *TP53* mutation across tumor types, with an overall accuracy of 91% to 97%; however, the interpretation parameters can vary widely.^47^, ^48^, ^49^ The possible mechanisms behind discordant immunophenotype and genotype for p53 include sample bias, tumor heterogeneity, and delayed p53 degradation.^49^, ^50^ In the present study, suboptimal fixation of some specimens may also have been a source of discordant staining since all p53 immunohistochemistry was performed on cytology specimens collected in alcohol‐based fixative. Three of the cases with increased p53 expression also had moderate to strong PD‐L1 expression in 50%–70% of tumor cells. Interestingly, these three cases all had missense mutations.

The association between *TP53* mutation and PD‐L1 expression has been established in prior studies.^51^, ^52^ However, a recent report has identified that only the missense type of *TP53* mutations was correlated with a better response to immune checkpoint inhibitors.^51^ The same study also showed an association between *TP53* missense mutation and other predictors of immunotherapy response, such as increased PD‐L1 levels, TMB, and neoantigen levels.^51^ The significant correlation between *TP53* missense mutation and PD‐L1 expression was likewise observed in the present study. Although statistically insignificant, increased TMB was observed in tumors with both missense mutation and truncated p53 protein. While these observations were limited by the small number of cases in this cohort, further clinical validation of these *TP53* associations is warranted for prognostic and clinical immunotherapy decisions. Assoun et al. investigated patients with advanced NSCLC who were treated with immune checkpoint inhibitors.^53^ They observed significantly longer progression‐free and overall survival among those patients with tumors harboring *TP53* mutations.

As mentioned, one limitation of this study is the small number of cases. Since the occurrence of isolated *TP53* mutation remains a rare event in lung adenocarcinoma, a multi‐institutional study may be necessary to expand the investigation on the clinical, therapeutic, and prognostic implications of an isolated *TP53* mutation in this lung tumor.

In summary, isolated *TP53* mutation is a rare event in lung adenocarcinoma. This sole pathogenic molecular event was seen in association with smoking, poor cytomorphologic features, adverse prognosis, and recurrent *CEBPA* deletions. Our findings suggest that *TP53* mutation alone may have the capacity to act as an oncogenic driver in lung adenocarcinomas, and its presence in isolation may be sufficient to trigger poor prognostic features in lung tumors. While these tumors tend to have strong PD‐L1 expression and high TMB, especially those with missense type of *TP53* mutation, prospective studies with higher sample size are necessary to further explore the possibility of a favorable response to immune checkpoint inhibitors and other targeted treatments. Hence, the recognition of this unique molecular group in lung adenocarcinomas has prognostic and therapeutic implications.

## AUTHOR CONTRIBUTIONS

**Rachelle P. Mendoza:** Data curation (lead); formal analysis (lead); investigation (equal); methodology (equal); writing – original draft (equal); writing – review and editing (equal). **Heather I‐Hsuan Chen‐Yost:** Conceptualization (equal); data curation (lead); formal analysis (equal); investigation (equal); methodology (lead); writing – original draft (lead); writing – review and editing (equal). **Pankhuri Wanjari:** Data curation (equal); formal analysis (equal); methodology (equal); validation (equal). **Peng Wang:** Data curation (equal); formal analysis (equal); investigation (equal); methodology (equal); validation (equal); writing – review and editing (equal). **Emily Symes:** Data curation (equal); methodology (equal); writing – review and editing (equal). **Daniel N. Johnson:** Conceptualization (equal); data curation (equal); supervision (equal); writing – review and editing (equal). **Ward Reeves:** Conceptualization (equal); methodology (equal); supervision (equal); writing – review and editing (equal). **Jeffrey Mueller:** Conceptualization (equal); project administration (equal); supervision (equal); writing – review and editing (equal). **Tatjana Antic:** Conceptualization (equal); data curation (equal); investigation (equal); methodology (equal); project administration (equal); supervision (equal); visualization (equal); writing – review and editing (equal). **Anna Biernacka:** Conceptualization (lead); data curation (equal); formal analysis (equal); investigation (equal); methodology (equal); project administration (lead); resources (lead); supervision (lead); validation (lead); visualization (equal); writing – review and editing (lead).

## FUNDING INFORMATION

This study has been internally funded by the Department of Pathology, University of Chicago.

## CONFLICT OF INTEREST STATEMENT

The authors have disclosed that they have no significant relationships with, or financial interest in, any commercial companies pertaining to this article.

## PRECIS FOR USE IN THE TABLE OF CONTENTS

In this study, lung adenocarcinomas with isolated *TP53* mutation were extensively characterized, providing the largest series to date on this rare group of tumors. This sole pathogenic molecular event in lung adenocarcinoma was seen in association with smoking, adverse cytomorphologic features, and poor prognosis.

## Supporting information


Figure S1.

Table S1.

## Data Availability

The data that support the findings of this study are available from the corresponding author upon reasonable request.
