# Supplementary figures and images for: Lung adenocarcinomas with isolated TP53 mutation: A comprehensive clinical, cytopathologic and molecular characterization
Source: Cancer Med. 2024 Jan 2;13(1):e6873. doi: 10.1002/cam4.6873 (PMC10824142; doi:10.1002/cam4.6873)

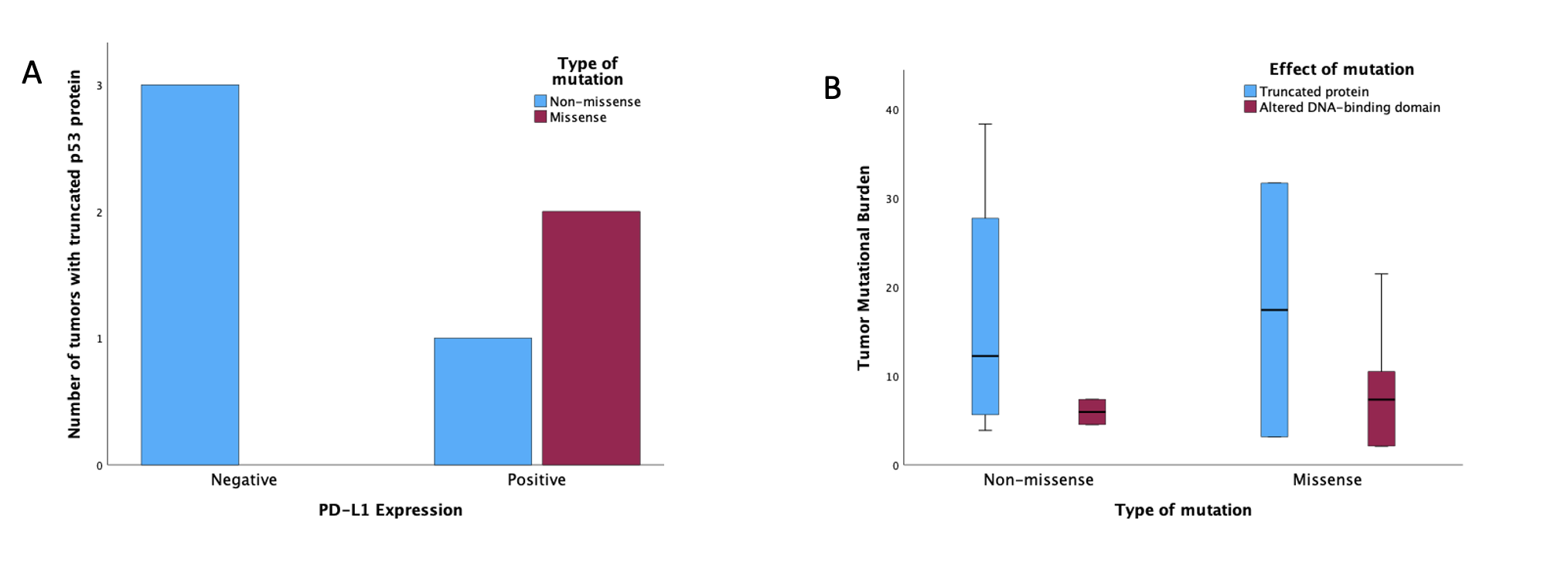

Supplement: Supplementary file 1 — Figure S1. [file CAM4-13-e6873-s002.png]
