# Supplementary material for: Lung adenocarcinomas with isolated TP53 mutation: A comprehensive clinical, cytopathologic and molecular characterization
Source: Cancer Med. 2024 Jan 2;13(1):e6873. doi: 10.1002/cam4.6873 (PMC10824142; doi:10.1002/cam4.6873)
Supplement: Supplementary file 2 — Table S1. [file CAM4-13-e6873-s001.docx]

**SUPPLEMENTAL TABLE**

Supplemental Table 1. Clinical, immunohistochemical, and molecular features of isolated *TP53*-mutated tumors based on the type of mutation.

| Characteristics | Categories | Type of mutation | | Type of mutation grouped according to effect of mutation | | | | | |
| --- | --- | --- | --- | --- | --- | --- | --- | --- | --- |
|  |  | Non-missense | Missense | Non-missense | | Missense | | | |
|  |  |  |  | Effect of Mutation | | Effect of Mutation | | | |
|  |  |  |  | Truncated Protein | Altered DNA-binding domain | | Truncated Protein | Altered DNA-binding domain |  |
| Age, years, average (range) | | 67.7 (48 - 79) | 64.3 (53 - 72) | 70.0 (62 - 79) | 63.0 (48 - 78) | | 62.5 (60 - 65) | 64.8 (53 - 72) |  |
| Sex | Female | 1 (20.0) | 4 (80.0) | 1 (100.0) | 0 (0.0) | | 2 (50.0) | 2 (50.0) |  |
|  | Male | 5 (55.6) | 4 (44.4) | 3 (60.0) | 2 (40.0) | | 0 | 4 |  |
| Laterality Coded | Right | 4 (50.0) | 4 (50.0) | 3 (75.0) | 1 (25.0) | | 1 (25.0) | 3 (75.0) |  |
|  | Left | 2 (33.3) | 4 (66.7) | 1 (50.0) | 1 (50.0) | | 1 (25.0) | 3 (75.0) |  |
| Tumor size, cm, average (range) | | 4.0 (1.3 - 8.1) | 3.5 (1.4 - 6.4) | 3.8 (1.3 - 8.1) | 4.5 (3.6 - 5.3) | | 4.0 (1.5 - 6.4) | 3.4 (1.4 - 6.4) |  |
| Recurrence | No | 3 (50.0) | 3 (50.0) | 2 (66.7) | 1 (33.3) | | 1 (33.3) | 2 (66.7) |  |
|  | Yes | 2 (33.3) | 4 (66.7) | 1 (50.0) | 1 (50.0) | | 1 (25.0) | 3 (75.0) |  |
| Status at follow-up | Alive | 2 (50.0) | 2 (50.0) | 1 (50.0) | 1 (50.0) | | 1 (50.0) | 1 (50.0) |  |
|  | Deceased | 4 (44.4) | 5 (55.6) | 3 (75.0) | 1 (25.0) | | 1 (20.0) | 4 (80.0) |  |
| Progression-free survival, months, median (range) | | 7.2 (3.3 - 64.3) | 11.8 (0.0 - 84.8) | 7.2 (3.3 - 12.5) | 35.2 (6.2 - 64.3) | | 42.4 (0.0 - 84.8) | 11.8 (2.6 - 36.6) |  |
| Overall survival, months, median (range) | | 9.0 (3.3 - 64.3) | 34.1 (6.3 - 101.4) | 7.1 (3.3 - 52.2)^a^ | 37.1 (9.8 - 64.3) | | 85.1 (68.8 - 101.4)^a^ | 12.2 (6.3 - 36.6) |  |
| p53 expression | Wild type | 5 (100.0) | 0 (0.0) | 4 (80.0) | 1 (20.0) | | 0 (0.0) | 0 (0.0) |  |
|  | Overexpressed | 1 (14.3)^b^ | 6 (85.7)^b^ | 0 (0.0) | 1 (100.0) | | 1 (16.7) | 5 (83.3) |  |
| PD-L1 expression | Negative | 4 (44.4) | 5 (55.6) | 3 (75.0) | 1 (25.0) | | 0 (0.0)^c^ | 5 (100.0)^c^ |  |
|  | Positive | 2 (40.0) | 3 (60.0) | 1 (50.0) | 1 (50.0) | | 2 (66.7)^c^ | 1 (33.3)^c^ |  |
| Microsatellite instability (MSI) score, average (range) | | 3.3 (1.8 - 5.4) | 3.0 (1.8 - 8.0) | 4.2 (2.4 - 5.4) | 1.8 (1.8 - 1.8) | | 3.4 (3.0 - 3.9) | 4.3 (1.8 - 8.0) |  |
| Tumor mutational burden (TMB), mut/Mb, average (range) | | 13.1 (3.9 - 38.4) | 7.3 (2.1 - 31.7) | 12.2 (3.9 - 38.4) | 5.9 (4.5 - 7.4) | | 17.4 (3.2 - 31.7) | 8.5 (2.1 - 21.5) |  |

a. Overall survival in tumors with truncated p53 protein was significantly shorter in tumors with non-missense mutation (p=0.005); b. Missense mutation was significantly correlated with p53 overexpression (p=0.003); c. Tumors with missense mutation and truncated protein had significantly higher PD-L1 expression (p=0.035).
